# Effectiveness of a digital rehabilitation program based on computer vision and augmented reality for isolated meniscus injury: protocol for a prospective randomized controlled trial

**DOI:** 10.1186/s13018-023-04367-3

**Published:** 2023-12-07

**Authors:** Li Wang, Xi Chen, Qian Deng, MingKe You, Yang Xu, Di Liu, Ye Lin, PengCheng Li, Jian Li

**Affiliations:** 1 Department of Orthopaedics, Orthopaedic Research Institute, West China Hospital, Sichuan University, No 37 Guo Xue Xiang, Chengdu, 610041 Sichuan People’s Republic of China; 2 Jiakang Zhongzhi Technology Company, Beijing, People’s Republic of China; 3 University of Chicago, Chicago, USA; 4 China School of Nursing, Sichuan University, Chengdu, People’s Republic of China

**Keywords:** Computer vision, Augmented reality, Meniscus, Rehabilitation

## Abstract

**Background:**

The lack of access to physical therapists in developing countries and rural areas poses a significant challenge in supervising postsurgical rehabilitation, potentially impeding desirable outcomes following surgical interventions. For this reason, this study aims to evaluate the feasibility, safety, and effectiveness of utilizing a digital rehabilitation program based on computer vision and augmented reality in comparison with traditional care for patients who will undergo isolated meniscus repair, since to date, there is no literature on this topic.

**Methods:**

This study intends to enroll two groups of participants, each to be provided with informed consent before undergoing randomization into either the experimental or control group. The experimental group will undergo a digital rehabilitation program utilizing computer vision and augmented reality (AR) technology following their surgical procedure, while the control group will receive conventional care, involving in-clinic physical therapy sessions weekly. Both groups will adhere to a standardized rehabilitation protocol over a six-month duration. Follow-up assessments will be conducted at various intervals, including preoperatively, and at 2 weeks, 6 weeks, 12 weeks, and 24 weeks postoperatively. Imaging assessments and return-to-play evaluations will be conducted during the final follow-up. Clinical functionality will be assessed based on improvements in International Knee Documentation Committee (IKDC) and Visual Analog Scale (VAS) scores.

**Registration number:**

ChiCTR2300070582.

## Introduction

The lateral and medial menisci protect articular cartilage by providing shock absorption, distributing load, and lubricating the articular surface. Injury to the meniscus is prevalent among young and athletic individuals, causing biomechanical alterations of the joint, resulting in increased joint contact stress and possibly precipitating early degenerative changes and osteoarthritis [1, 2]. Studies have established that meniscectomy increases the likelihood of accelerated knee osteoarthritis; thus, arthroscopic procedures with suturing or meniscoplasty (central meniscal resection) are usually employed to preserve as much meniscal tissue as possible, depending on the shape of the damaged meniscus [1–4].

Postoperatively, physical therapy plays a pivotal role [5]. However, a significant challenge arises from the limited access to skilled therapists in developing countries and remote rural areas [6–8]. Patients in these resource-constrained regions often face long commutes to healthcare facilities, reduced compliance with rehabilitation protocols, and a lack of monitoring during the postoperative phase [6]. Consequently, recognizing the limitations inherent in conventional clinic-based physical therapy practice, digital rehabilitation programs will emerge as a promising supplemental solution. [7, 9]

Advanced technologies such as augmented reality and computer vision will have the potential to offer interactive digital therapy experiences [10]. Currently, the utilization of the VR system facilitates home-based rehabilitation training and increases its interactivity and enjoyment. [11–15] However, in VR, patients interact with a virtual environment that simulates real-life activities. The risk associated with this technology is that potentially dangerous situations may not be appropriately identified; however, images portrayed in both AR and virtual reality overlap with real-world images, thus allowing patients to be aware of potential dangers. [10] The incorporation of both virtual and real-world elements combined with real-time interaction and standard rehabilitation protocols can be leveraged to promote the recovery of an injured joint [15]. Telerehabilitation using technologies such as computer vision (CV) offers the potential for improving access to rehabilitation programs. The use of markerless human pose estimation based on computer vision in telerehabilitation is a promising research area, as it offers the advantage of closely monitoring movement without the need for external markers to capture motion data [16]. In our study, AR technology will utilize readily available devices such as smartphones, eliminating the need for additional hardware and providing greater convenience, while computer vision will facilitate real-time monitoring of patient movements and exercise postures.

The protocol of the digital rehabilitation program is based on the concept of accelerated rehabilitation after meniscal surgery proposed in 1996, suggesting that early postoperative weight bearing and knee range of motion (ROM) could reduce the risk of joint adhesions and muscle atrophy [5]. In recent years, accelerated rehabilitation programs for early weight bearing and active ROM after meniscus repair have shown positive results in patients with longitudinal meniscus tears [1, 2, 17]. Further research is warranted to investigate the safety and efficacy of early weight bearing and ROM training in patients who undergo complex meniscal tear repair, as the rehabilitation strategies may vary depending on tear pattern. [2, 18]

As a result, this study will utilize computer vision and smartphone-based augmented reality to overcome the limitations associated with conventional postoperative rehabilitation methods, with the goal of enhancing orthopedic postoperative outcomes. This study will involve delivering standardized exercise protocols using a digital platform, providing patients with continuous and effective physical therapy training supplemented by real-time feedback. Notably, there is currently no literature reporting the application of augmented reality and computer vision for postoperative recovery in patients with isolated meniscus injuries.

## Aim of study

This study will recruit adult participants diagnosed with isolated longitudinal meniscus injuries confirmed through arthroscopic examination and scheduled for meniscus repair. The primary objective of this research is to assess the safety, effectiveness, and feasibility of implementing a digital rehabilitation program that incorporates computer vision and augmented reality as part of the postoperative rehabilitation process.

## Methods

### Study design

**Fig. 1:**
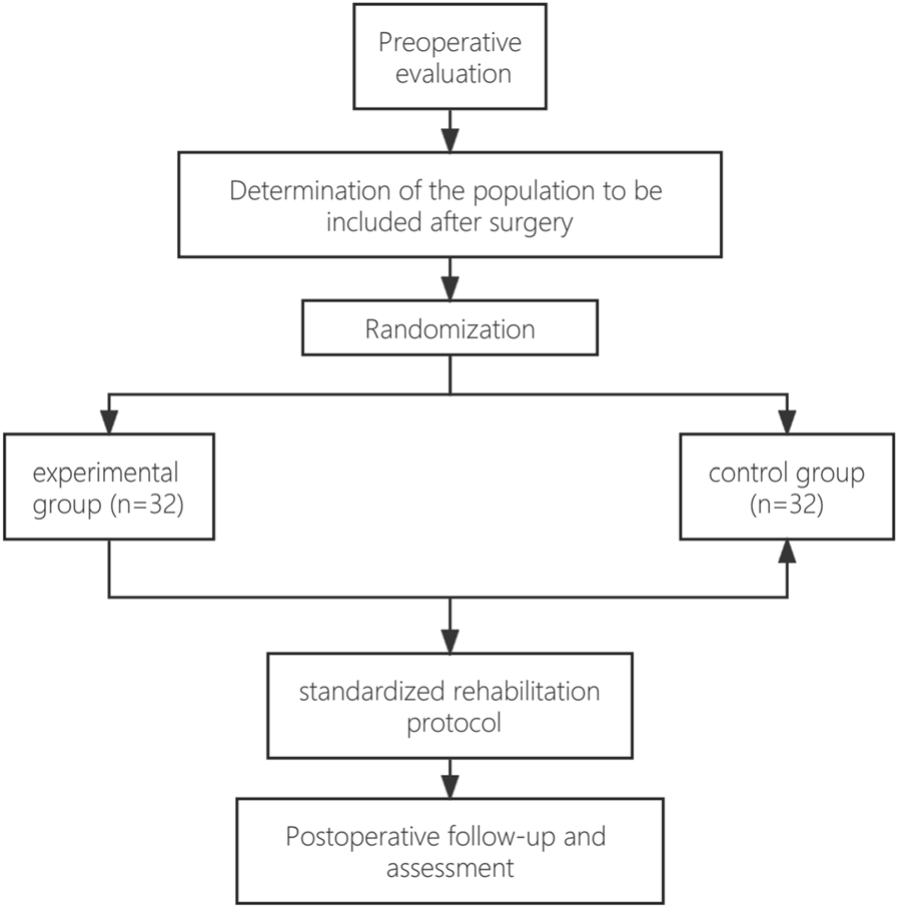
Research flowchart

This is a single-center, prospective, randomized controlled study. After receiving informed consent, participants will be randomly allocated into two groups: the experimental group and the control group will adopt the standardized rehabilitation protocol (Table 1). The experimental group will engage in a digital rehabilitation program that can be completed from home, whereas the control group will attend weekly physical therapy clinic sessions for exercise guidance. The flowchart of the study is shown in Fig. 1.

**Table 1:** The standardized postoperative rehabilitation plan

Overall goal: Stage 1: 0–2 weeks: Control of swelling and symptoms after surgery, early progressive weight bearing, and ROM
Control of swelling and symptoms: (1) apply ice at least 3 times a day after surgery, 20 min each time; (2) apply the elastic bandage to compress the affected limb; (3) elevate the affected limb and lie flat on your back when sleeping and cushion a pillow with a certain thickness under the affected limb
ROM and brace use: (1) 0–60 degree fixation, early gentle knee movement under sitting position (2) heel support training, ensuring knee joint at 0 degrees
Weight bearing: The knee brace is secured in the fully extended knee position, and crutches are used to transition from no weight bearing to partial weight bearing as tolerated
Exercises: (1) sitting hamstring pull (2) ankle pump (3) quadriceps isometric training (4) straight leg elevation training (15–30 degrees) (5) hamstring isometric training
Overall goal: Stage 2:3–6 weeks: Early exercise and strength training, ROM, and partial weight bearing as tolerated
ROM and support use: (1) 3–4 weeks: 0–90-degree fixation, complete knee extension and bend to 90 degrees (2) 5–6 weeks 0–120-degree fixation, bend to 120 degrees
Weight bearing: With the knee brace fixed in the fully extended knee position, and using crutches, gradual weight bearing to full weight under tolerable conditions, and gradually remove of the crutches
Exercise: In addition to the training in the previous stage, include: (1) knee shin slide (2) supine straight leg raise 30 to 60 degrees (3) hip outreach training (4) lift heel stand (5) hip outreach (6) hip and (7) seat after knee flexion and stand
Overall goal: Stage 3: Weeks 7–12: Functional regression, gradual reduction of brace use, and restoration of total joint range of motion
ROM and brace use: (1) restore full joint range of motion and (2) gradually stop brace use after full weight bearing
Exercise: Add advanced resistance training: (1) recumbent resistance straight leg raise (2) step-down training (3) heel raise training (4) knee flexion (5) straight leg draft with elastic band (6) recumbent resistance heel slide (7) forward lunge (8) half squat against wall 50 degrees (9) four-point kneeling cross stretch
Overall goal: Stage 4: Weeks 13–24: Early exercise training, full-strength recovery, cardiovascular exercise adaptation, and exercise-specific training (speed and agility training)
Exercise: Continue with the third stage of training and build on this cardiovascular endurance training (power cycling, recovery running, and jumping)
Phase 5: after return to sports assessment to determine whether to return to body contact, rotating sports

### Randomization

SPSS 23.0 (IBM, New York, NY, USA) will be used to generate random numbers. Subjects with odd numbers will be assigned to the experimental group, and subjects with even numbers will be assigned to the control group. The envelope method will be used for hidden grouping, which will be implemented by a third party. The random allocation scheme will be stored in an opaque envelope, which will be opened in succession according to the order of inclusion, thereby determining the assigned group for each patient. After that, the envelopes will be given to the study implementer, who will then use inclusion and exclusion criteria to decide whether each patient can be included in the study.

### Blinding method

For this study, blinding of the interveners and patients was not feasible. Blinding should be applied to the data collectors, data analysts, and outcome assessors in the study.

### Subjects

Inclusion criteria:

1. Isolated meniscus injury diagnosed by magnetic resonance imaging (MRI)
2. Confirmation of a longitudinal tear pattern (including bucket handle tears) under arthroscopy, with a repairable tear.
3. Suture meniscus repair with or without partial meniscectomy
4. Participants have sustained their meniscus injury due to physical activities, such as playing basketball.
5. Willing to participate in this clinical trial and receive follow-up.

Exclusion criteria:

1. Preoperative MRI diagnosis with other ligament or chondral lesions;
2. A discoid meniscus was diagnosed under arthroscopy.
3. Trauma and surgery to other weight-bearing joints in the lower extremities, such as torn ligaments in the ankle and necrosis of the femoral head in the hip, can indirectly affect knee load and movement.
4. Knee osteoarthritis, defined by Kellgren–Lawrence grade II or higher or Outerbridge classification grade II and above. [19, 20]

### Sample size

The sample size is calculated based on the calculation method of a pilot study [21], and the effect size is calculated as follows: *d* = 0.8; *α* = 0.05; Power = 0.8. The minimum sample size per group is 26, and the estimated loss of follow-up/exit rate is 20%. Thus, a maximum of 32 participants will be recruited for each group.

### Intervention measures

#### Surgical procedure

All surgeries will be performed by 1 designated surgeons from our center. These designated surgeons are required to undergo a meniscal surgical technique evaluation and register with the center before being permitted to participate in the study and perform surgeries. Arthroscopic surgery will be performed under general anesthesia. If repairable meniscal lesions are found in the patient, the lesions will be first cleared using a shaver for freshening. All patients will receive a full-endoscopic meniscal repair technique, and if the meniscus is not repairable, the partial meniscectomy can be performed to reshape the meniscus.

#### Postoperative rehabilitation protocol

A standardized postoperative rehabilitation protocol will be adopted according to the clinical guidelines in the American Academy of Orthopaedic Surgeons (AAOS) postoperative rehabilitation manual18 and previous studies1,2,4,14 (Table 1). [1, 2, 4, 18, 22]

#### Experimental group

**Fig. 2:**
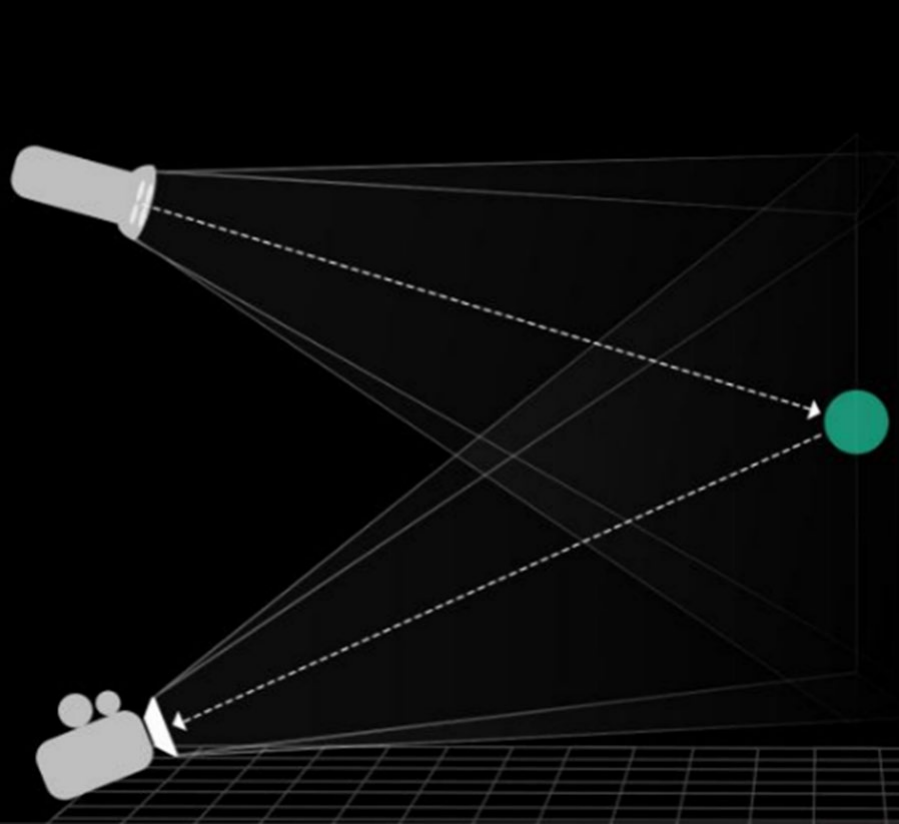
The camera system

Patients will receive daily exercise plans via a software platform for 12 weeks. This software can be installed on the patients' smartphones. Once the patient initiates the digital therapy platform, the system will utilize the smartphone's camera to capture the patient's position in real time (Fig. 2). As the patient performs the physical therapy exercises, the system will provide demonstration videos, verbal and auditory feedback on the patient's exercise posture and duration. The digital platform will also record the patient's exercise frequency and duration. This data will then be transmitted to the software platform for detailed analysis by the research team.

#### Smartphone camera requirements

It is recommended to have a photo resolution of 1280 × 720 pixels or higher and a video frame rate of 30 frames per second or higher for the smartphone camera.

#### Environment

The environment in which the camera is placed should have appropriate lighting conditions. Adequate natural light or suitable illumination can enhance image quality and recognition accuracy. The camera should be placed in a stable position to avoid image blurring. The camera's position should be adjusted to capture the patient in the frame. Ensure that the camera's field of view is unobstructed.

#### Human pose detection model

**Fig. 3:**
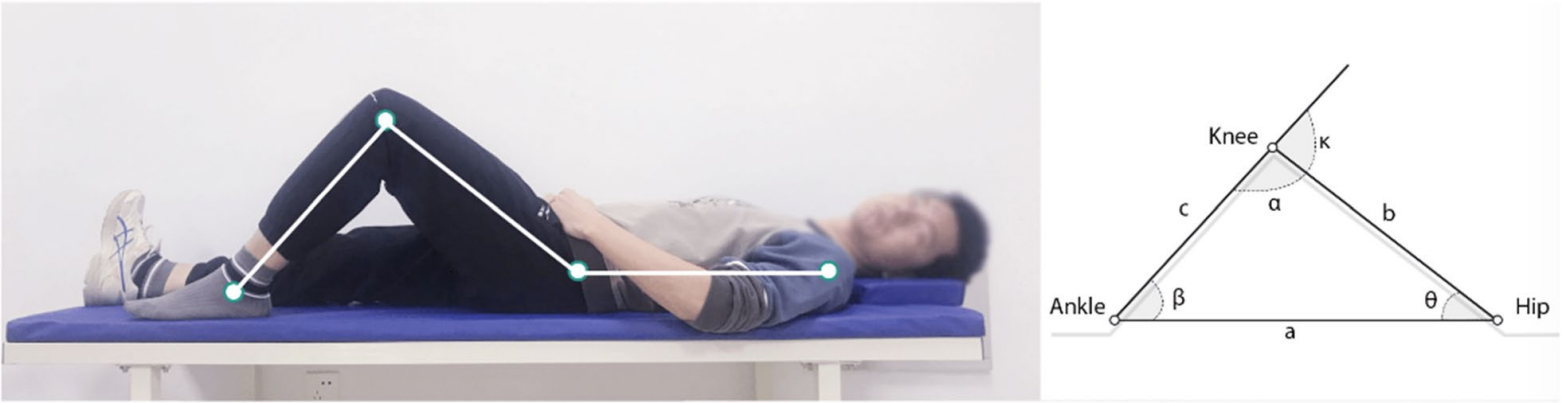
Human pose detection model recognizing knee flexion

**Fig. 4:**
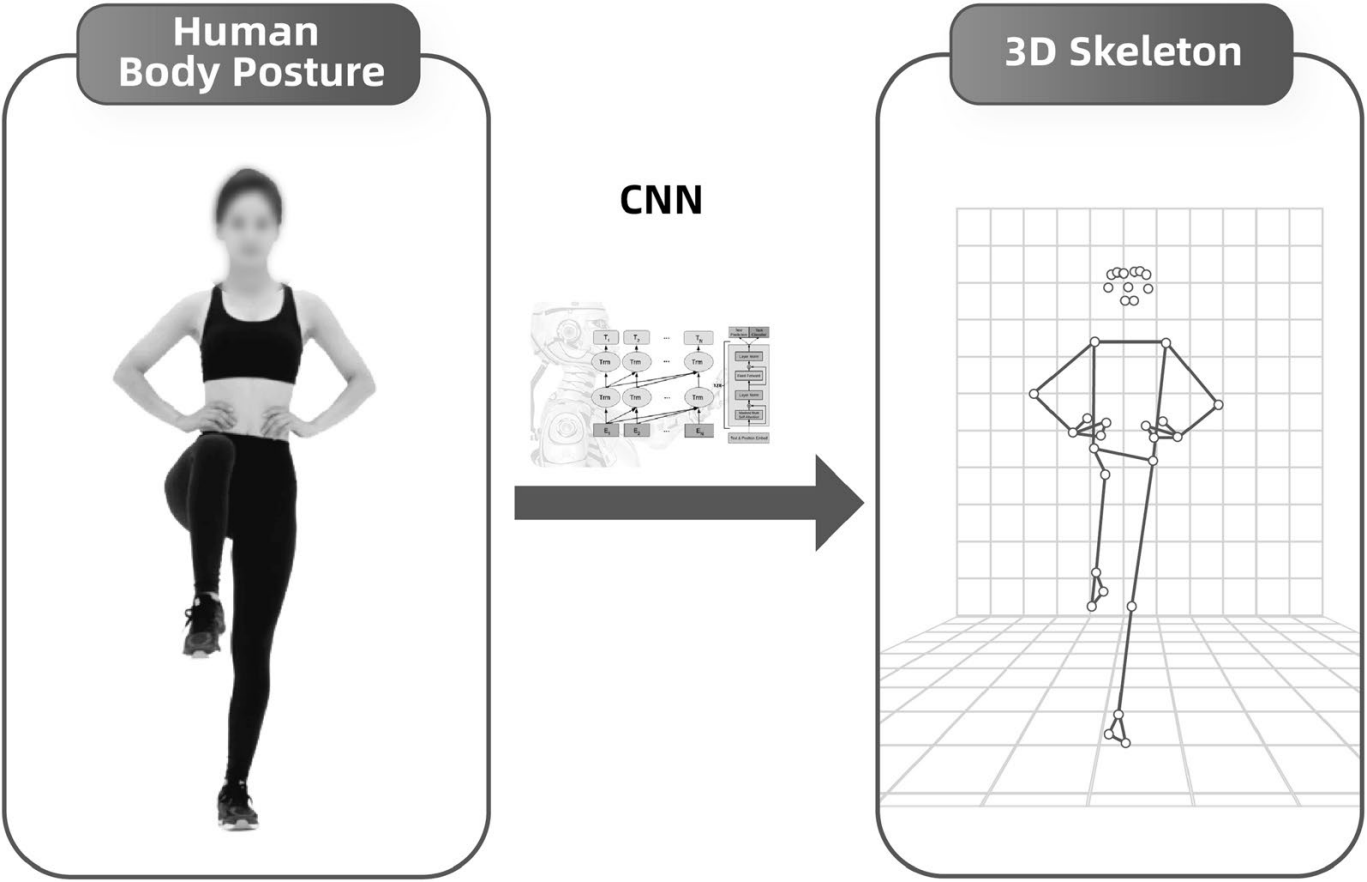
3D skeletal model reconstruction

The human pose detection model (Fig. 3) will detect critical anatomical landmarks on the human body using images or videos captured by a smartphone camera. By employing a convolutional neural network (CNN) algorithm, this model can identify various joints, including the shoulder, elbow, wrist, hip, knee, and ankle. It will then generate a 3D skeletal model (Fig. 4), tracking dynamic changes in the patient's body position during motion.

When the model detects exercise postures that deviate from preset values, it will provide auditory and verbal feedback via the software to remind the patient to adjust their position based on the instructional video. Hence, the patient will receive interactive feedback during the training process.

#### Control group

Patients will be referred to the clinic weekly for 12 weeks postoperatively, during which they will receive instructions on physical therapy exercises. Every week, patients will learn the exercises at the clinic and will then be asked to take the instruction sheet home to complete the remaining exercises.

#### Compliance

To increase compliance, patients will receive adequate education on software usage before the start of the trial. Throughout the trial, patients will receive detailed instructions via handouts and reminders via texts and calls. Support will be provided to patients to answer questions throughout the trial. Compliance data will be monitored based on software usage data and patient attendance data in the clinic. The research team will collect data on patients' training frequency and duration to objectively assess participant compliance within the study.

### Outcomes

#### Clinical knee function evaluation

The estimated follow-up time was 1 day before surgery and 2, 6, 12, and 24 weeks after surgery, and the specific evaluation items included knee ROM (2, 6, 12, 24 weeks after surgery), weight-bearing progress (when fully weight bearing) and knee function and pain scores [23] (Lysholm score, Tegner score, IKDC knee subjective function score, and VAS score). At the final follow-up assessment, return-to-play (RTP) status will be evaluated based on the rehabilitation literature available for meniscus repair. **(**Table 2**)**. [4, 24, 25]

**Table 2:** Return-to-play assessment

VAS score = 0	
IKDC score > 90	
No active effusion (Brush test negative)	
The difference in circumference between the quadriceps muscles was less than 1.5 cm	
The knee isokinetic strength test showed a ratio of bilateral quadriceps muscles greater than 90%, bilateral hamstrings greater than 90%, and unilateral hamstrings to quadriceps muscle greater than 66%	
Jump test Limb symmetry index (LSI = jump distance on the affected side/jump distance on the healthy side *100%) was greater than 90%, and the test items included a single-leg long jump test and a single-leg triple jump test	
Y-word balance test: bilateral extension asymmetry within 4 cm; bilateral comprehensive score is greater than 90%	
Lateral step-down test showed no dynamic genu valgus	

The clinical function of patients was assessed and evaluated based on IKDC and VAS score improvement via the criteria of minimal clinically important difference (MCID), patient accepted symptom state (PASS), and significant clinical benefit (SCB) [25].According to the study of Gowd et al. [25], the MCID threshold, which represents the minimal clinically important difference, is defined as an improvement of at least 10.6 points in the IKDC score after treatment or intervention. This threshold signifies that a clinically meaningful change in knee joint function is recognized. The SCB threshold, indicating significant clinical benefit, is set at an IKDC score of 27.3 points or higher after treatment or intervention. This threshold is used to confirm that the treatment has brought about a significant clinical benefit. Lastly, the threshold for patient accepted symptom state (PASS) is established at an IKDC score of 57.9 points or higher. This threshold signifies that patients subjectively consider their symptom state to be acceptable.

#### Radiographic evaluation

Evaluation time point and project: X-ray: 1 day before surgery, 1 day, and 6 months after surgery; the anteroposterior and lateral position of the knee joint was examined; MRI (T1-weighted and T2-weighted images of axial, coronal and sagittal positions): 1 day before surgery, 6 months after surgery. The imaging assessment was conducted in a blinded manner, and the evaluators were not informed of the identity and grouping of the patients. Imaging evaluation should be performed without intervention.

#### Complications assessment

Postoperative complications, such as postoperative knee infection, deep venous thrombosis of the lower limbs, stiffness, and arthrofibrosis, will be recorded.

## Statistical analysis

Quantitative data will be analyzed using SPSS 21.0 (IBM, New York, NY, USA). The demographic, social, and preclinical characteristics of the subjects in both groups will be described. Differences in these variables between the intervention and control groups will be analyzed using either the Chi-square test or the *T* test, with continuous variables recorded as the mean and standard errors and categorical variables as rates (incidence). For continuous variables, the Shapiro‒Wilk test will be performed to verify that the variables in each group obeyed a normal distribution. For repeated measures, 2-way ANOVA analysis will be conducted. Two independent samples mean comparison *T* tests were used to compare the normally distributed continuous variables between groups. For nonparametric variables, the Kruskal‒Wallis test was used to determine the differences between the groups. Chi-square tests and Fischer exact tests were used to determine differences in categorical variables. Statistical significance was set at *p* < 0.05.

## Adverse event management

In this study, inadequate healing of the meniscus may be observed following suturing. After a 6-month postoperative MRI review and clinical evaluation, some patients may require an arthroscopic review to confirm meniscus healing. If the repair of the meniscus is unsuccessful, then partial meniscectomy will be undertaken.

## Data Availability

The datasets used or analyzed during the current study are available from the corresponding author upon reasonable request.
